# PoPMuSiC 2.1: a web server for the estimation of protein stability changes upon mutation and sequence optimality

**DOI:** 10.1186/1471-2105-12-151

**Published:** 2011-05-13

**Authors:** Yves Dehouck, Jean Marc Kwasigroch, Dimitri Gilis, Marianne Rooman

**Affiliations:** 1 Bioinformatique génomique et structurale, Université Libre de Bruxelles, Av. Fr. Roosevelt 50, CP165/61, 1050 Brussels, Belgium

## Abstract

**Background:**

The rational design of modified proteins with controlled stability is of extreme importance in a whole range of applications, notably in the biotechnological and environmental areas, where proteins are used for their catalytic or other functional activities. Future breakthroughs in medical research may also be expected from an improved understanding of the effect of naturally occurring disease-causing mutations on the molecular level.

**Results:**

PoPMuSiC-2.1 is a web server that predicts the thermodynamic stability changes caused by single site mutations in proteins, using a linear combination of statistical potentials whose coefficients depend on the solvent accessibility of the mutated residue. PoPMuSiC presents good prediction performances (correlation coefficient of 0.8 between predicted and measured stability changes, in cross validation, after exclusion of 10% outliers). It is moreover very fast, allowing the prediction of the stability changes resulting from all possible mutations in a medium size protein in less than a minute. This unique functionality is user-friendly implemented in PoPMuSiC and is particularly easy to exploit. Another new functionality of our server concerns the estimation of the optimality of each amino acid in the sequence, with respect to the stability of the structure. It may be used to detect structural weaknesses, i.e. clusters of non-optimal residues, which represent particularly interesting sites for introducing targeted mutations. This sequence optimality data is also expected to have significant implications in the prediction and the analysis of particular structural or functional protein regions. To illustrate the interest of this new functionality, we apply it to a dataset of known catalytic sites, and show that a much larger than average concentration of structural weaknesses is detected, quantifying how these sites have been optimized for function rather than stability.

**Conclusion:**

The freely available PoPMuSiC-2.1 web server is highly useful for identifying very rapidly a list of possibly relevant mutations with the desired stability properties, on which subsequent experimental studies can be focused. It can also be used to detect sequence regions corresponding to structural weaknesses, which could be functionally important or structurally delicate regions, with obvious applications in rational protein design.

## Background

The availability of computational tools yielding reasonably accurate estimations of the impact of amino acid substitutions on the stability of proteins is of crucial importance in a wide range of applications. In particular, such tools have the potential to stimulate and support protein engineering and design projects dedicated to the creation of modified proteins that remain active in non-physiological conditions, or that present enhanced functional properties [1,2]. On the other hand, advances in the ability to predict and rationalize the functional effect of naturally occurring amino acid variants and their relationship to disease will have tremendous implications in medicine. Indeed, they can be expected to lead to significant improvements in the understanding of the mechanisms of various diseases, to the development of enhanced diagnostics, new therapeutic approaches, and more personalized treatment options [3,4]. Although approaches based on multiple sequence alignments remain predominant in this context, predictions of stability changes upon mutation have been recognized as a relevant input in the identification of deleterious and disease-causing mutations [4-7]. On a more fundamental level, the analysis of the predicted distributions of stabilizing or destabilizing mutations in sets of natural or engineered proteins may be extremely valuable to refine our understanding of the relationships between protein sequence, structure, and function [8-11], or to probe the evolutionary dynamics of protein sequences [11-14].

Over the last decade, several methods have been developed to predict the effects of mutations on the stability of proteins. Many of these methods rely primarily on an energy function describing the interactions between residues, within a simplified structural representation. We may distinguish the approaches based on statistical potentials extracted from datasets of protein structures [15-19], from those based on empirical potentials built from optimised combinations of various physical energy terms [20-23]. Several predictors were set up with the help of machine learning technologies, through the establishment of an empirical relationship between the stability change upon mutation and a large number of sequence and/or structural features of the mutated and mutant amino acids [24-27]. More recently, approaches combining the advantages of statistical energy functions and machine learning tools have also been described [28,29].

Three recent studies independently assessed and compared the performances of several of those predictors, using datasets of experimentally characterized mutants that had not been used to train any of the predictive models [22,29,30]. Overall, the conclusions were mixed: all methods show a correct trend in the predictions, but the accuracies often remain moderate. PoPMuSiC was however shown to be a standout and to perform quite well in comparison with several other methods [29]. It should be considered that most of these methods are extremely fast with respect to more detailed approaches, such as free energy perturbation [31] or thermodynamic integration [32]. In particular, PoPMuSiC allows the estimation of the stability changes resulting from all possible point mutations in an average-sized protein in a matter of seconds.

This advantage was exploited to predict the stability changes induced by all possible point mutations in several globular proteins [10], using the FoldX algorithm [20]. The results were in good agreement with previous experimental studies, in that a large majority of mutations appear to have a destabilizing effect on protein structures. Moreover, the overall distributions of predicted stability changes were shown to be very similar in different globular proteins. However, it has to be stressed that although this trend holds true - on average - for whole proteins, some local regions may present a different behavior. For example, residues belonging to the active site of a protein have been selected during evolution so as to ensure proper functioning, and are thus generally less optimal with respect to stability [33,34]. Specific protein regions associated with peculiar patterns of stability changes upon mutation corresponding to structural weaknesses were also suggested, using the PoPMuSiC algorithm, to be involved in the occurrence of conformational changes, such as 3D domain swapping [35] or amyloid fibril formation [15]; the latter structural weaknesses were supported by experimental analyses [36].

We present here the PoPMuSiC-2.1 web server, which allows fast and accurate predictions of the stability changes resulting from point mutations in globular proteins. Besides its top-level performances, our server also distinguishes itself from other available tools by an important advantage in terms of computational speed, and by the ability to perform a systematic scan of all possible mutations in a protein. A new functionality of the current PoPMuSiC web server is that it gives the opportunity to obtain easily an estimation of the optimality of each residue in a protein's sequence, with respect to the stability of its structure. To illustrate the general interest of this unique feature, we performed a large-scale investigation of the optimality of residues involved in catalytic sites, and discuss the possibility of using such data to improve methods aiming at predicting functional sites in proteins.

## Implementation

### Prediction of protein stability changes upon mutations

The stability change resulting from a given point mutation in a protein is computed on the basis of the structure of the wild-type protein and a set of energy functions, which are used to estimate the folding free energy change upon mutation of a residue *s_w _*into *s*_m_, noted ΔΔ*G*_P_(*s*_w_, *s*_m_). More precisely, ΔΔ*G*_P _is expressed as a linear combination of 13 statistical potentials (ΔΔ*W*_i_, *i *= 1, 13), two terms that depend on the volume of the wild-type and mutant amino acids (Δ*V*_±_), and an independent term:(1)

The coefficients α_i _depend on the solvent accessibility *A *of the wild-type amino acid *s*_w_. The potentials Δ*W*_i _are derived from a dataset of known protein structures and describe the correlations between various sequence or structure descriptors of the same amino acids or of neighboring ones, according to the previously described formalism [37]. The descriptors considered are, for each residue: the amino acid type *s*, the torsion angles defining the backbone conformation *t *and the solvent accessibility *a*, and, for each pair of residues: the spatial distance between the average geometric centers of their side chains *d*. The 13 potentials Δ*W*_i _are denoted in terms of these descriptors as Δ*W*_st_, Δ*W*_as_, Δ*W*_sd_, Δ*W*_sds_, Δ*W*_stt_, Δ*W*_sst_, Δ*W*_aas_, Δ*W*_ass_, Δ*W*_ast_, Δ*W*_asd_, Δ*W*_std_, Δ*W*_asdas_, Δ*W*_stdst_. The terms of the type Δ*W*_vw _and Δ*W*_vwx _are defined as:(2)

where *v*, *w*, *x *are any of the descriptors *s*, *t*, *a *and *d*, *k *is the Boltzmann constant and *T *the absolute temperature. The terms Δ*W*_asdas _and Δ*W*_stdst _are defined in a similar way [37]. Higher order coupling terms are not taken into account, since they were shown to yield no improvement in the prediction of stability changes upon mutation [29]. In addition to the statistical potentials, two terms in eq.(1), *i.e*. Δ*V*_±_, are related to the volume difference between the mutant and wild-type amino acid: Δ*V = V*_m_*-V*_w_. They are defined as Δ*V*_± _= Δ*V *H(± ΔV), where the H is the Heaviside function. They provide a coarse description of the impact of creating of a cavity (if Δ*V*<0) or accommodating a larger side-chain within the protein structure (if Δ*V*>0). Statistical potentials cannot be expected to describe correctly such effects, since they are derived from a dataset of native structures of wild-type proteins, with very few packing defects.

The weighting coefficients α_i_(*A*) (*i *= 1, 16) were chosen to be sigmoid functions of the solvent accessibility (*A*) of the mutated residue:(3)

where *c*_i _is the inflection point of sigmoid *i*, *r*_i _its slope, *f*_i _its scaling factor and *b*_i _its vertical shift. The reason for this choice is that it enables the description of a smooth transition between two different environments: the protein core and the protein surface. Indeed, it was shown previously that the relative weights of the different types of interactions vary according to whether they concern residues at the surface or in the core [38].

Our predictive model thus includes 64 different parameters (16 *c*_j_, 16 *r*_j_, 16 *f*_i_, and 16 *b*_i_). The values of these parameters were estimated with the help of a neural network model minimizing the mean square error (σ) on the ΔΔ*G *predictions for a dataset of *N *experimentally characterized protein mutants [29]:(4)

where ΔΔ*G*_M, m _is the experimentally measured folding free energy change of mutant *m *and ΔΔ*G*_P, m _its predicted value, obtained with eqs. (1)-(3). An iterative parameter reduction procedure was devised to eliminate the parameters that present a large uncertainty, which reduced their number from 64 to 52 [29].

**Figure 1:**
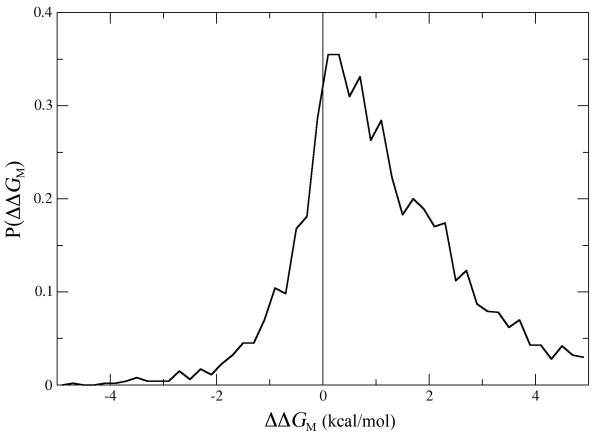
**Distribution of the measured values of ΔΔ*G *in the dataset of 2648 mutants used to train and validate PoPMuSiC**.

The dataset used to train and validate the model contains 2648 different single-site mutations, in 131 proteins of known structure, whose impact on the folding free energy of the protein has been experimentally determined [29]. The data was originally extracted from the the ProTherm database [39], and thoroughly checked to correct or eliminate erroneous inputs. Mutations introduced in heme proteins or in pseudo-wild type constructs were not considered. Mutations that involve a proline or destabilize the structure by more than 5 kcal/mol were also rejected, since they are likely to induce structural modifications that are not taken into account by PoPMuSiC. The distribution of the measured changes in folding free energy caused by the mutations that are present in our dataset is given in Figure 1, and is very similar to previously published and discussed distributions of free energy changes upon mutations [10].

### Estimation of protein sequence optimality

PoPMuSiC is fast enough to estimate within seconds the stability changes resulting from all possible mutations in an average-sized protein. It is therefore possible to estimate how robust the structure of a given protein is against mutations in its sequence. It is also possible to identify positions that are particularly poorly optimized with respect to protein stability, *i.e*. positions for which the predictions suggest that several possible mutations would improve stability. The ability to identify such positions in a protein sequence may be of substantial interest. Indeed, they obviously constitute attractive targets for protein engineering applications. They may also be involved in the mechanisms of protein function [33], the occurrence of structural switches or the development of conformational diseases [15,35].

For each position *i *in the sequence of a protein, we define a score Γ*_i _*that quantifies the degree of non-optimality of the amino acid at this position, with respect to the overall stability of the protein:(5)

where H is the Heaviside function, *m *is one of the 19 possibilities of mutation of the amino acid *w *in position *i*, and ΔΔ*G*_P,*w*i→*m *_is the corresponding predicted stability change. The score Γ is thus the sum of the predicted stability changes of all stabilizing mutations at a given position in the sequence. Since the large majority of mutations have a destabilizing effect on the protein, Γ can be expected to be close to zero for many positions in the sequence. In contrast, very negative values of Γ point out particularly interesting positions, where some mutations are strongly stabilizing and/or many mutations mildly stabilizing.

### Web interface

PoPMuSiC is called by a user-friendly PHP/MySQL web interface. Since the predictions of PoPMuSiC are based on the structure of the target protein, all queries require a structure file to be specified. The user may either provide the 4-letter code of the Protein DataBank (PDB) structure, which will then be automatically retrieved from the PDB server [40], upload his own structure file, or select a previously uploaded file. The user may choose to provide a structure file generated by a modeling approach, as long as it complies with the PDB format. Note, however, that the performances of PoPMuSiC were evaluated on the basis of experimentally resolved protein structures and are likely to be lower for modeled structures. Obviously, the accuracy of the predictions will depend on the quality of the model.

Three types of queries may be performed:

• The "Single" query allows the prediction of the stability change resulting from one given mutation, specified by the user, in the protein of interest.

• The "File" query allows the stability change prediction of a list of single-site mutations in a protein of interest. A (plain text) file containing the list of mutations must be uploaded. The server will output a (plain text) file containing the predicted stability change resulting from each mutation.

• The "Systematic" query allows the prediction of the stability changes resulting from all possible single-site mutations in the protein of interest. The server will output a (plain text) file containing the predicted stability change resulting from each mutation. The user may choose how the results will be ordered: either sequentially or on the basis of the value of the predicted ΔΔ*G_P_*s.

**Figure 2:**
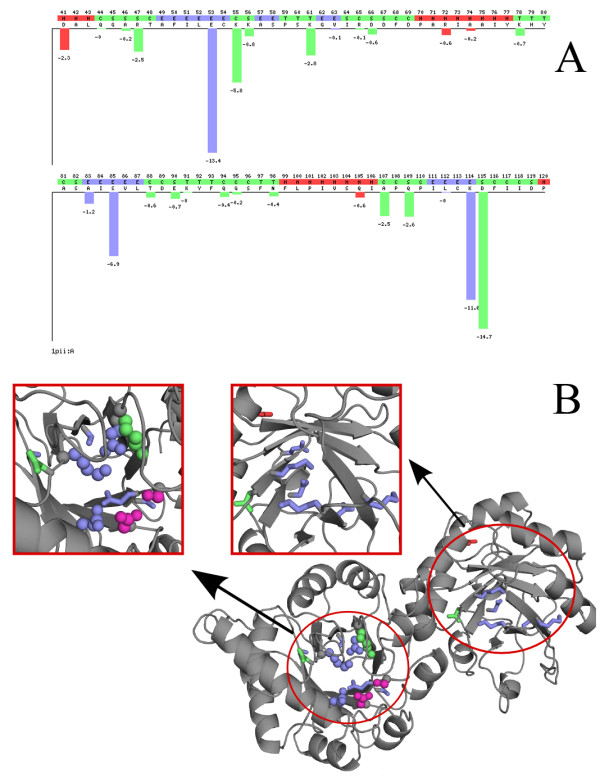
**Sequence optimality in the active site of the PRAI-IGPS enzyme.** A. Sample output of the PoPMuSiC-2.1 web server, corresponding to residues 41-120 of the bifunctional enzyme PRAI:IGPS from E. Coli (PDB code: 1PIIpdb1PII). The sequence optimality score Γ is plotted as a function of the position in the sequence. The elements of secondary structure are distinguished by the associated colour: helices in red, strands in blue, and coils in green. B. Schematic representation of the PRAI:IGPS enzyme. The residues identified by PoPMuSiC as being non-optimal with respect to the stability (Γ ≤ -5 kcal/mol) are highlighted in red (helix), blue (beta strand), or green (coil region). The residues recorded as catalytic residues in the Catalytic Site Atlas are represented as spheres, while those identified by PoPMuSiC but not recorded in the Catalytic Site Atlas are represented as sticks. Catalytic residues that are not identified by PoPMuSiC are colored in magenta.

The sequence optimality scores (Γ) are automatically computed for each "Systematic" query. A second plain text file, containing the Γ-values for each position in the sequence is then given as output. In addition, an interactive figure is created, which allows the user to view the distribution of Γ-values along the sequence, and to easily identify the individual contribution of each mutation (Figure 2A).

## Results and Discussion

### Comparison of predicted and measured stability changes

**Figure 3:**
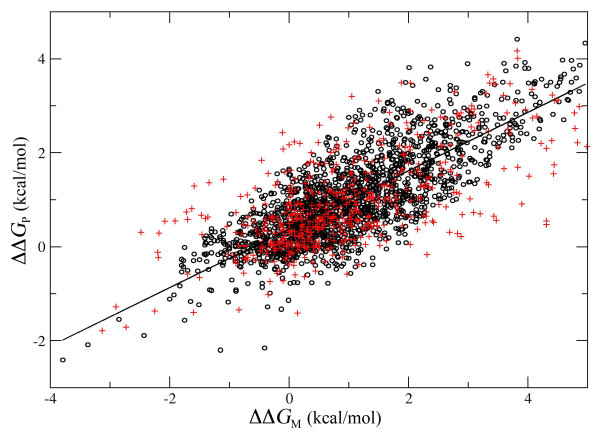
**The predicted stability changes (ΔΔ*G*_P_) are plotted against the corresponding measured values (ΔΔ*G*_M_) for the 2648 mutants of our dataset, after one of the 5 runs of cross-validation**. Mutations belonging to the training set are depicted in black (o), while mutations belonging to the validation set are depicted in red (+).

The performances of PoPMuSiC in predicting the changes in folding free energy resulting from single-site mutations were evaluated using a 5-fold cross validation procedure [29]. In a first step, the values of the parameters of the α_i_(*A*) functions (eq. (3)) were identified so as to minimize the root mean square error between predicted and measured ΔΔ*G *values (eq. (4)) on a learning set containing 4/5 of the whole dataset of 2648 mutants, chosen at random. In a second step, these parameter values were applied to predict the ΔΔ*G_P _*values for the test set containing the remaining 1/5 of the dataset. Five different runs were performed, so that every 1/5 of the dataset was considered once as test set and that each mutant was included once in a test set. A graphical comparison of the measured values of ΔΔ*G *with those predicted during one of these five runs is given in Figure 3, for both the training and the validation set. The Pearson correlation coefficient *R *and the root mean square error σ (eq. (4)) between measured and predicted stability changes, in the training and validation sets, are reported in Table 1 for each of these five runs. The results from direct validation, where the parameters were identified and the predictions performed on the learning set containing the same 4/5 of the data, are also given for sake of comparison.

**Table 1:** Performances in 5-fold validation

	All mutants		Exclusion of 10% outliers	
	
	*R*_d_/*R*_c_^a^	σ_d_/σ_c _(kcal/mol)^b^	*R*_d_/*R*_c _^a^	σ_d_/σ_c _(kcal/mol)^b^
ΔΔ*G*_P _**= <**ΔΔ*G*_M _**>**	-	1.47	-	1.10
Random predictor^c^	0.00 (0.02)	2.08 (0.02)	0.22 (0.02)	1.60 (0.02)

Run 1	0.64/0.62	1.13/1.16	0.79/0.78	0.86/0.89
Run 2	0.63/0.64	1.15/1.08	0.79/0.78	0.87/0.85
Run 3	0.65/0.59	1.11/1.20	0.80/0.78	0.85/0.88
Run 4	0.64/0.61	1.12/1.18	0.80/0.77	0.85/0.88
Run 5	0.64/0.63	1.13/1.15	0.79/0.78	0.85/0.89
**Average 1**^d^	**0.64/0.62**	**1.13/1.16**	**0.79/0.78**	**0.85/0.88**
**Average 2**^d, e^	**-/0.63**	**-/1.15**	**-/0.79**	**-/0.86**

^a ^Correlation coefficient between predicted and measured ΔΔ*G's*, in the training (*R*_d_) and validation (*R*_c_) set. ^b ^root mean square error between predicted and measured ΔΔ*G's*, in the training (*R*_d_) and validation (*R*_c_) set. ^e ^The random predictor is obtained by using a randomly shuffled set of ΔΔ*G*_M _values as predicted ΔΔ*G *values. The average (standard deviation) values of *R *and σ over 1000 runs are given. ^d ^Average values of *R *and σ, over the five different runs. ^e ^Badly modeled mutants are removed from the training sets before parameter identification, but they are maintained in the validation sets.

As expected, the performances are slightly better in direct validation (on the training set) than in cross validation (on the validation set), but the differences are quite small, indicating the absence of overfitting. On average, the correlation coefficient *R*_c _between predicted and measured ΔΔ*G *values is 0.62, and the root mean square error σ_c _is 1.16 kcal/mol. These measures of performance indicate a strong improvement over the random predictor, which uses randomly shuffled ΔΔ*G*_M _values as predicted ΔΔ*G*_P _values, and yields on average a root mean square error of 2.08 kcal/mol (Table 1). Setting all ΔΔ*G*_P _values equal to the average of the ΔΔ*G*_M _values generates a root mean square error of 1.47 kcal/mol (Table 1), and is thus more efficient than the random predictor, but still far from reaching the performances of PoPMuSiC. The predictive power of PoPMuSiC was also shown to surpass that of five previously published prediction tools, on an independent dataset of 350 mutations. Indeed, these five methods yielded a value of *R*_c _comprised between 0.29 and 0.48, as compared to 0.67 for PoPMuSiC, and a root mean square error σ_c _comprised between 1.43 and 4.12 kcal/mol, as compared to 1.16 kcal/mol for PoPMuSiC [29]. A recently published prediction method, PEAT-SA, was also benchmarked using the same dataset of 350 mutations: a *R*_c _value of 0.5 and a root mean square error of 1.92 kcal/mol were reported [23].

The values of *R *and σ after removal of the 10% most badly predicted mutations are also reported in Table 1. These values provide relevant complementary information to the performance indicators computed on the whole dataset, since a number of poorly predicted mutations may be related to experimental measurements made in specific, non-physiological, conditions or affected by a significant error, to a poorly resolved structure, to mistakes in the database indexing of the measured ΔΔ*G *value, or to structural modifications that are not taken into account by PoPMuSiC.

The last row of Table 1 corresponds to a second round of parameter identification, performed after removal from the training sets of all mutants for which |ΔΔ*G*_P_−ΔΔ*G*_M_| is larger than 1.5 kcal/mol in each of the five initial runs. The validation sets are left unchanged, in order to obtain comparable results. This induces a slight improvement of the performances in cross-validation, indicating that the presence of outliers in the training set had a negative impact on the identification of the model.

**Figure 4:**
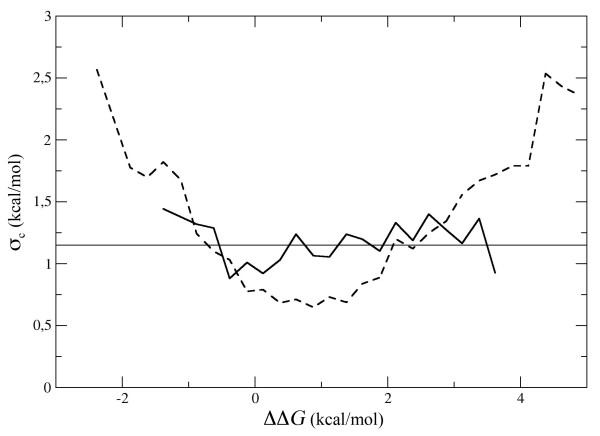
**The root mean square error between predicted and measured ΔΔ*G *values in the validation sets (σ_c_) is given as a function of ΔΔ*G*_P _(continuous line) and ΔΔ*G*_M _(dashed line)**. The dataset of mutations was divided in non-overlapping subsets corresponding to intervals of ΔΔ*G*_P _(or ΔΔ*G*_M_) of 0.25 kcal/mol width, and σ_c _was computed on each subset separately using eq. 4. Subsets containing less than 10 mutations were dismissed.

It is also interesting to know whether the precision of the predictions depends on the actual value of the free energy change. On average, PoPMuSiC performs better on mutations that fall in the most populated range of ΔΔ*G*_M _values, i.e. -0.5 kcal/mol < ΔΔ*G*_M _< 2.0 kcal/mol (Figure 4). As could be expected, the error is higher on mutations with an uncommonly strong stabilizing (ΔΔ*G*_M _< -2.0 kcal/mol) or destabilizing (ΔΔ*G*_M _> 4.0 kcal/mol) effect. However, it is important to notice that, from the point of view of the user, it is the predicted value of the free energy change (ΔΔ*G*_P_) that matters. As can bee seen on Figure 4, the error on the predictions does not show any clear dependency with respect to ΔΔ*G*_P_.

A frequent objective of protein engineering studies is the increase of the structural stability of a protein, via the introduction of mutations in its sequence. For such applications, PoPMuSiC can be used to identify a small subset of mutations that are likely to present the desired properties, and can be tested experimentally. An important indicator of the performances would then be the proportion of mutations in this subset that actually stabilize the structure, which is related to the specificity of the predictions with respect to stabilizing mutations. In this regard, PoPMuSiC appears as a very reliable prediction tool. Indeed, in cross-validation, 70% of the mutations predicted as mildly stabilizing (ΔΔ*G*_P _< -0.5 kcal/mol), and 86% of the mutations predicted as strongly stabilizing (ΔΔ*G*_P _< -1.0 kcal/mol), are actually stabilizing (ΔΔ*G*_M _< 0.0 kcal/mol).

The good performances of PoPMuSiC were made possible by using a combination of statistical potentials, whose relative weights were optimized via a simple neural network. The total number of adjusted parameters is 52, which remains very reasonable with respect to the size of the training/validation dataset (2648 non-redundant entries), and with respect to other prediction methods based on machine learning techniques [24-28]. Our model also clearly distinguishes itself from a pure black box, as the overall shapes of the optimized weighting functions were shown to exhibit little dependence on the training set, and possess biophysical significance [29].

### Speed of the computations

In addition to its high level of performances, and in particular its good specificity to stabilizing mutations, the ability of the PoPMuSiC-2.1 server to rapidly process all possible mutations in a protein is a very significant practical advantage over competing algorithms. Indeed, as shown in Table 2, PoPMuSiC is currently the only prediction tool that allows a systematic scan of all possible mutations via a single, simple, web-based query. Furthermore, the overall speed of the predictions is one to several orders of magnitude larger than that of other web servers. These unique features make PoPMuSiC-2.1 an instrument of choice for obtaining support and guidance in the design of protein engineering experiments.

**Table 2:** Comparison of the practical features of different web servers

	Maximal number of mutations per query^a^	Number of mutations processed per minute^d^		
		
		1ag2 (*N *= 103)	3mbp (*N *= 370)	1gog (*N *= 689)
PoPMuSiC-2.1	19 × *N *^b^	6700	9000	4300
AutoMute [28]	19 × 5	13	4	1
CUPSAT [18]	19	600	600	600
D-mutant [17]	19	50	14	8
Eris [21]	1	< 0.1	< 0.1	< 0.1
I-mutant2.0 [24]	19^c^	8	7	6
Hunter [22]	1	0.4	0.3	0.2
PEAT-SA [23]	*N *^b^	40	44	41

^a ^*N *is the number of residues in the target protein. ^b ^These web servers also allow the submission of a mutation list. ^c ^This program is also available for download, which allows the creation of scripts for batch computations. ^d ^Each presented value was obtained after averaging over three queries submitted to the corresponding web server. Obviously, the speed of the computations may depend on the load of the server at the time of the query. The presented values should therefore not be viewed as anything more than rough estimations.

### Case study: sequence optimality scores in catalytic sites

To illustrate the relevance and usefulness of the sequence optimality scores Γ computed by PoPMuSiC-2.1, we investigated the relationship between the involvement of residues in protein function and their nonoptimality with respect to protein stability. More precisely, we computed the Γ-score for each residue in a dataset of proteins whose catalytic sites have been experimentally identified and reported in the literature. Our analysis is based on version 2.2.10 of the Catalytic Site Atlas [41]. We selected only the "original" entries, *i.e*. proteins for which evidence of the location of the catalytic site comes directly from literature references, and excluded the "homologous" entries found by sequence alignment to one of the original entries. The resulting dataset contains 964 proteins, with 3227 catalytic residues and approximately 3.7 10^5 ^other residues.

Table 3 shows that the proportion of catalytic residues for which the computed optimality score Γ is lower than a given threshold is consistently much larger than the corresponding proportion for all other residues. In addition, the distinction between the catalytic residues and the others is more pronounced when a more stringent threshold value is chosen: *e.g*. with a threshold value of -1 kcal/mol, the proportion of nonoptimal residues is about two times larger in catalytic sites (28% versus 15%), whereas it is about four times larger with a threshold value of -5 kcal/mol (9% versus 2%).

**Table 3:** Γ optimality score for catalytic versus noncatalytic residues

Γ threshold (kcal/mol)	Catalytic residues^a^			Other residues^a^		
	All (N = 3227)	Core (N = 1784)	Surface (N = 1443)	All (N = 3.7 10^5^)	Core (N = 1.6 10^5^)	Surface (N = 2.1 10^5^)
Γ < 0.0	50.7%	52.6%	48.4%	38.2%	27.3%	46.3%
Γ < -0.25	39.4%	43.5%	34.4%	26.9%	21.1%	31.2%
Γ < -0.5	33.6%	38.5%	27.6%	21.3%	18.2%	23.7%
Γ < -1.0	27.7%	33.5%	20.7%	15.0%	14.4%	15.6%
Γ < -2.0	20.1%	26.3%	12.7%	8.8%	9.9%	8.1%
Γ < -3.0	15.1%	21.1%	7.8%	5.5%	7.1%	4.5%
Γ < -5.0	9.2%	14.0%	3.4%	2.4%	3.9%	1.5%
Γ < -7.5	5.1%	8.2%	1.4%	0.9%	1.9%	0.4%
Γ < -10.0	2.5%	4.4%	0.4%	0.4%	1.0%	0.1%

^a ^For each subset of our database, the fraction of residues whose optimality score Γ is lower than a given threshold is reported.

We also investigated the relationship between solvent accessibility and sequence optimality. For that purpose, the residues were distributed in two classes (Core and Surface) according to whether their relative solvent accessibility, computed with NACCESS [42], is smaller or larger than 10%. Table 3 indicates that the overall proportion of nonoptimal catalytic residues is larger in the core than on the surface of proteins (53% versus 48%, for Γ < 0 kcal/mol), and that this difference gets more striking when lower threshold values are considered (*e.g*. 14% versus 3%, for Γ < -5 kcal/mol). These results denote a stronger trade-off between stability and function in the core of proteins: selecting residue types at specific positions along the sequence to ensure proper functioning is on average more detrimental to protein stability when these residues have a low solvent accessibility. This can be related to the fact that many catalytic residues are charged and/or polar, and thus more likely to have a destabilizing impact when buried in the protein core.

In contrast, in the case of residues that are not involved in catalytic sites, the overall proportion of nonoptimal residues is quite larger on the surface than in the core (46% versus 27% for Γ<0 kcal/mol). It is however interesting to notice that this trend is inverted when threshold values of Γ lower than -2 kcal/mol are considered. These results are in good agreement with previously published studies, which reported that mutations of core residues are more likely to be detrimental to protein stability, while the distribution of stability changes induced by mutations on the surface is quite narrow, with very few highly (de)stabilizing effects [10]. It may also be related to the fact that surface residues have more often nonoptimal conformations because of crystal constraints or interaction with ligands.

Figure 2A is an example of the results obtained with PoPMuSiC on the bifunctional enzyme phosphoribuloseanthranilate isomerase:indoleglycerolphosphate synthase (PRAI:IGPS) from E. Coli (PDB code 1PII) [43]. According to the Catalytic Site Atlas, the residues of the N-terminal domain (residues 1-255) involved in the IGPS catalytic activity are Glu53, Lys55, Lys114, Glu163, Asn184, and Ser215. With a threshold value of -5 kcal/mol on the Γ-score, PoPMuSiC identifies only nine non-optimal residues in this domain, including four of the six catalytic residues. Interestingly, among the five other residues that present a Γ-value lower than -5 kcal/mol, four are located in the same cavity, in close contact with the catalytic residues (Figure 2B). They hold thus probably some importance with respect to the affinity or the specificity of the interaction with the ligand. In the C-terminal domain (residues 256-452) that catalyses the PRAI reaction, PoPMuSiC identifies 11 residues with a Γ-value lower than -5 kcal/mol. The catalytic residues of this domain are not recorded in the Catalytic Site Atlas. However, as can be seen on Figure 2B, many of the residues pointed out by PoPMuSiC are clustered together, in a region previously identified as the PRAI active site [43]. Overall, it appears thus that a large majority of the 20 residues identified as non-optimal by PoPMuSiC in this protein are involved in its enzymatic activity, even though only four of them were recorded in the Catalytic Site Atlas [41].

## Conclusions

We present a web server for the prediction of protein stability changes upon mutations and for the estimation of the optimality of each amino acid in a protein's sequence with respect to the stability of its structure. The prediction performances were evaluated by a 5-fold cross validation procedure, and turned out to be quite impressive for a coarse-grained and very fast prediction method: the correlation coefficient *R *between predicted and measured ΔΔ*G*s is 0.63 and the root mean square error σ = 1.15 kcal/mol. The performances increase up to *R *= 0.79 and σ = 0.86 kcal/mol after removal of 10% outliers. PoPMuSiC was also shown to outperform several other prediction tools, on an independent dataset of 350 mutations that were not included in the training sets of the compared methods [23,29].

A significant advantage of PoPMuSiC-2.1 is that it allows the rapid computation of the stability changes resulting from all single-site mutations in a protein. It can thus be used in the context of rational protein design, to help identify, among the multitude of possibilities, a small number of mutations that are likely to present the desired stability properties. For example, the different versions of PoPMuSiC [15,29] have been successfully applied by several groups (including us) to predict mutations in the prion protein that stabilize the soluble form and occur in a region that has since then been shown to be determinant for the aggregation tendencies [36], to modulate the polymerization propensity of α_1_-antitrypsin [44], to increase the solubility of a TEV protease by stabilizing the folded state relatively to the aggregated form [45], to stabilize the folded dark state of a photocontrolled DNA-binding protein in view of modulating the degree of photo-switching [46], or to identify mutations that stabilize various enzymes, such as pyruvate formate-lyase [47] or feruloyl esterase [48]. PoPMuSiC has also been used to characterize *in silico *the effect on stability of specific mutations, in view of rationalizing their impact on a protein of therapeutic interest. The considered mutations were for example naturally occurring variants responsible for the development of hereditary diseases [49-52], mutations related to the acquisition of drug resistance in bacteria [53], or spanning the natural genetic heterogeneity of a viral protein [54].

Another consequence of the speed of the predictions is that PoPMuSiC-2.1 can be used to evaluate the optimality of the sequence of a protein with respect to the stability of its structure. This optimality, which is the result of evolution, is shown to be intimately related to the mechanisms of protein function. We indeed applied our prediction method to a large number of enzymes whose catalytic sites have been previously identified and recorded in the Catalytic Site Atlas [41]. Our results indicate that catalytic residues are on average significantly less optimal than other residues, with respect to protein stability. Although the same general trend is observed both on the surface and in the core of proteins, it is much stronger in the core, which is in agreement with previous studies of protein stability.

Obviously, the distinction between catalytic and noncatalytic residues is not perfectly clearcut. According to our predictions, approximately half of the catalytic residues are nonoptimal with respect to protein stability, which means that the other half are totally optimal and thus that all possible mutations of these residues are predicted as destabilizing. This observation indicates that many residues playing an essential role in protein function are not detrimental to stability, which somewhat relativizes the well-known trade-off between stability and function [33,34,55]. On the other hand, a number of noncatalytic residues were also identified as not optimal with respect to stability. It is very likely that many of these residues are somehow involved in protein function without being actually part of the catalytic site. They may for instance be close to this site and important to ensure a proper binding affinity or specificity with a ligand, or to generate a sufficient level of structural flexibility [56,57]. The example of the PRAI:IGPS protein nicely illustrates this point, given that most of the nonoptimal residues highlighted by PoPMuSiC were clustered in the two active sites of this enzyme (Figure 2). Other nonoptimal residues may be present as a result of a compromise between protein stability and other constraints such as the kinetics of the folding or binding process, the prevention of misfolding [9], or the adjustment of the resistance to proteolysis [58]. Following previously published arguments, we may also note that evolution is a dynamic process, during which protein stability is kept in a near-optimal state by mutations that slightly diminish stability without causing any deleterious effect [14]. Finally, the predictions are of course not perfectly accurate, which may lead to incorrectly label some residues as being not optimal, especially in regions where the structure is poorly defined or subject to crystal constraints.

The inference of protein function is currently often performed through the analysis of sequence conservation data derived from multiple sequence alignments, despite the limitations inherent to this approach [59]. A number of other computational methods have also been developed to identify unknown functional sites in proteins on the basis of structural features [60-62]. These include geometry-based methods such as the detection of pockets or cavities that could accommodate a ligand, energy-based methods such as the identification of sites that interact favorably with various types of probes, and knowledge-based methods that typically involve structural comparisons with datasets or atlases of known functional sites. Some studies were also conducted to investigate the contribution of functional residues to the overall stability of the protein, sometimes with a predictive purpose [9,63-66]. Recent developments tend to be more focused on the design of prediction schemes integrating various types of information, such as structural attributes and evolutionary sequence conservation, in order to benefit from their complementary [67-69]. To our knowledge, the optimality of the amino acids with respect to protein stability is not explicitly taken into account by any of these integrated methods.

Although further studies are necessary to clarify the relationship between sequence optimality and protein function, our results strongly support the idea that the inclusion of sequence optimality data is likely to improve the performances of methods that aim at identifying unknown catalytic sites or other function-related residues. In addition, such sequence optimality data may be of interest in various types of other applications, such as the assessment of the quality of model protein structures, or the investigation of the evolutionary dynamics of proteins. This data may also provide complementary information to that derived from other prediction tools in view, for instance, of identifying hot spots for molecular recognition [70,71] or protein aggregation [72].

## Availability and Requirements

Project name: PoPMuSiC

Project home page: http://babylone.ulb.ac.be/popmusic

Operating system: Platform independent (web server)

Programming language: C, PHP/HTML

Any restrictions to use by non-academics: none

## Competing interests

The authors declare that they have no competing interests.

## Authors' contributions

YD and MR designed the study; YD and JMK implemented the web server; YD and DG performed the analysis of sequence optimality in catalytic sites; YD drafted the manuscript; YD, MR and DG finalized the manuscript. All authors read and approved the final manuscript.
